# Applicability of Selected 3D Printing Materials in Electrochemistry

**DOI:** 10.3390/bios12050308

**Published:** 2022-05-07

**Authors:** Marta Choińska, Vojtěch Hrdlička, Hana Dejmková, Jan Fischer, Luděk Míka, Eva Vaněčková, Viliam Kolivoška, Tomáš Navrátil

**Affiliations:** 1 J. Heyrovský Institute of Physical Chemistry of the Czech Academy of Sciences, Dolejškova 3, 182 23 Prague, Czech Republic; marta.choinska@jh-inst.cas.cz (M.C.); vojtech.hrdlicka@jh-inst.cas.cz (V.H.); eva.vaneckova@jh-inst.cas.cz (E.V.); viliam.kolivska@jh-inst.cas.cz (V.K.); 2 Department of Analytical Chemistry, Faculty of Science, Charles University, Albertov 6, 128 00 Prague, Czech Republic; hana.dejmkova@natur.cuni.cz (H.D.); jan.fischer@natur.cuni.cz (J.F.); 3 Department of Chemistry Education, Faculty of Science, Charles University, Albertov 6, 128 00 Prague, Czech Republic; ludek.mika@natur.cuni.cz

**Keywords:** 3D printing materials, mechanical stability, chemical stability, electrochemistry, cyclic voltammetry, differential pulse voltammetry, anodic stripping voltammetry

## Abstract

This manuscript investigates the chemical and structural stability of 3D printing materials (3DPMs) frequently used in electrochemistry. Four 3D printing materials were studied: Clear photopolymer, Elastic photopolymer, PET filament, and PLA filament. Their stability, solubility, structural changes, flexibility, hardness, and color changes were investigated after exposure to selected organic solvents and supporting electrolytes. Furthermore, the available potential windows and behavior of redox probes in selected supporting electrolytes were investigated before and after the exposure of the 3D-printed objects to the electrolytes at various working electrodes. Possible electrochemically active interferences with an origin from the 3DPMs were also monitored to provide a comprehensive outline for the use of 3DPMs in electrochemical platform manufacturing.

## 1. Introduction

The application of 3D printing is steadily spreading into electrochemistry due to the efficiency it provides to the manufacturing of cells, electrode parts, and even the electrodes themselves, particularly in low-scale production [1]. The printing process is precise, easy, fast, often inexpensive, safe, and allows for versatile production and product modifications [2,3,4,5,6,7].

Various 3D printing techniques have been developed over time. The most common is fused deposition modeling (FDM), based on the protruding a fiber of the thermoplastic material through a heated nozzle [4,5,8]; the melted extrusion is deposited on the growing object in a specified pattern [4,8,9,10]. The most popular thermoplastic materials are polylactic acid (PLA), acrylonitrile butadiene styrene (ABS), acrylonitrile styrene acrylate (ASA), nylon and other polyamides, polypropylene (PP), polycarbonate, or polyetherimide [2,9,11]. Polymers can also be mixed with another material before forming into the filament. This process might help to reinforce the material, but the possibility to add an electrically conductive component (e.g., graphite or graphene) to obtain a conductive printing material [1,7,12,13] is particularly interesting for electrochemistry. Another common 3D printing method is stereolithography (SLA), in which a liquid polymer precursor is photopolymerized using UV or visible radiation. The light beam moves according to the project instructions and polymerizes the material. Alternatively, a diode array as a light source is used for digital light processing (DLP) with the irradiation carried out by particular switchable pixels in the diode field [14,15,16]. SLA uses printing materials with radical polymerization, such as acrylates, vinyl ethers, or epoxy resins. Often a mixture of the various monomers is used to reach the desired properties of the final polymeric material. In comparison with FDM, SLA provides structures with a better resolution, but it cannot combine several printing materials during the printing process [14,17,18]. The disadvantage of SLA and DLP is that printed objects require rinsing by organic solvents, which makes the manufacturing process costly and waste-producing.

Regardless of the used printing technique, its application for electrochemistry requires sufficient chemical stability of the employed materials with regard to the printing conditions and minimum amount of soluble, electrochemically active interferences. Choosing suitable 3D printing materials (3DPMs) to print electrochemical cells and systems is essential to avoid their undesirable changes because the properties of the materials may be affected by long-term exposure to various common solvents, chemical compounds, or extreme pH values [19,20]. The damage or disintegration of the printed objects is the usual result of a careless application; however, some of these processes can be utilized to pretreat or regenerate electrodes by the selective etching of the polymeric material. On the other hand, the printing material can affect the electrochemical properties of the electrolyte/electrode system, e.g., the influence on the oxygen or hydrogen evolution reactions. In addition, the chemical compatibility may differ in the cases of original virgin filament, the printed object, and may even be different from supplementary information provided by the manufacturers and vendors, as shown by Heikkinen et al. [21]. In any case, interactions between the 3DPMs and chemicals used during the experiments should be understood and characterized before printing an object or starting an experiment.

So far, the properties of composite electrodes with glassy carbon fibers [19], PLA [22], and ABS [23] were mainly explored regarding their dependence on the used printing parameters [24,25], fiber modification [26,27], and post-printing treatment [22,28,29,30]. While properties of 3D printed electrodes have been investigated extensively, significantly less attention was paid to insulating materials to be used for other cell parts.

This paper focuses on the comparison of the stability of four 3DPMs and the change of potential windows of electrolytes after 24 h of contact with these materials. The four materials were chosen as the most popular ones for fabrication of particular parts of an electrochemical cell or experimental platform: PLA as a cheap, biodegradable, and the most common material for fabrication of conductive parts (mixed with carbon or metallic additives), PET as a cheap and durable material for electrically non-conductive parts, and two resins: Elastic for the fabrication of mechanically stressed parts and Clear for the transparent parts. The investigation includes the physical changes of these filaments and photopolymerized resins after exposure to alkaline, acidic, and organic solutions [31].

Furthermore, changes in potential windows in such electrolytes after exposure to the 3DPMs are compared using hanging mercury, glassy carbon, and 3D-printed PLA/carbon black electrodes. Finally, the influence on peak potentials and peak currents of model analytes or redox probes after exposure to these 3DPMs will be investigated at the selected electrodes. This will provide a better understanding of the various effects of 3DPMs in electrochemical experiments, allowing a knowledge-based way to select a proper fiber or resin for the desired application.

## 2. Experimental

### 2.1. 3D Printed Tested Objects

Four non-conductive 3DPMs were used for the exposure tests. SLA resins were: Elastic (Formlabs, Somerville, MA, USA)—the resin of acrylate monomer, and Clear (Formlabs, Somerville, MA, USA)—a resin made of a mixture of dimethacrylate, methacrylate monomer, and diphenyl(2,4,6-trimethylbenzoyl)phosphine oxide; FDM filaments were PLA Extrafill TrafficWhite (Fillamentum, Hulín, Czech Republic) and EPR InnoPET Natural (Innofil3D, Emmen, Netherlands).

**Figure 1:**
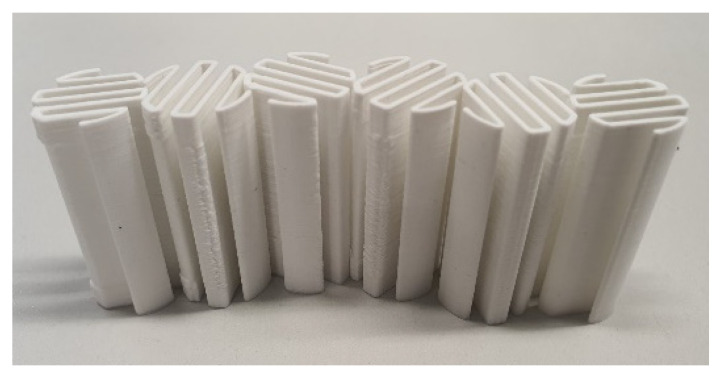
3D printed models from TrafficWhite PLA.

The tested objects for the mechanical stability testing were prepared in the shape of a square plate (dimensions 10 × 10 × 1 mm) and for the voltammetric measurement in the shape of a “7-wall accordion” on a circular base (diameter 15 mm, height 30 mm, wall width 1.0 mm), as shown in Figure 1. The accordion shape was chosen to maximize the surface to study possible electrochemically active interferents that can be dissolved from selected material.

The FDM printer was an original Prusa i3 MK3S+MMU2S (Prusa research, CZ) with Spring Steel Sheet with Smooth Double-sided PEI and E3D V6 0.4 nozzle (0.4 mm diameter, all Prusa research, CZ). Printing parameters for PET and PLA were 200 μm layer thickness, bed temperature 60 °C, and extruder temperature 215 °C and 210 °C for the first and the following layers, respectively. No brims or additional treatment after printing were used for the FMD-printed objects.

Stereolithographic resins Elastic and Clear were used in the SLA printer Formlabs Form 3 (Formlabs, USA), with manufacturer recommended settings: 405 nm LED light, intensity 1.25 mW cm^−2^, and 100 μm layer thickness. Afterward, they were cleaned two times with isopropanol, left to dry under the ambient temperature, and hardened in Formlabs Cure (Formlabs, USA) at 60 °C for 30 and 20 min, respectively.

### 2.2. Chemicals

The solvents used for solubility and stability tests were: acetone, acetonitrile (ACN), chloroform, cyclohexanone, dichloromethane, diethylether, dimethyl sulfoxide, ethanol, ethyl acetate, formaldehyde, hexane, isopropanol, methanol (MeOH), 1-penthanol, tetrahydrofuran, toluene, (all Penta, Prague, Czech Republic), and N,N-dimethyl formamide (Sigma Aldrich, MO, USA).

Aqueous 1 mol dm^−3^ solutions of inorganic electrolytes were prepared from H_2_SO_4_, NaOH, and KCl (all Lachema, Brno, Czech Republic). A mixed aqueous–organic electrolyte was prepared from 1 mol dm^−3^ KCl and MeOH (1:9, *v/v*).

K_3_[Fe(CN)_6_], Cd(NO_3_)_2_ (both Lachema, Brno, Czech Republic), and [Ru(NH_3_)_6_]Cl_3_ (Sigma Aldrich, Prague, Czech Republic) (stock solution concentration 1 × 10^−3^ mol dm^−3^) were used as model redoxactive probes. All chemicals were of p.a. purity grade, and aqueous solutions were prepared using Milli-Q-Gradient water (Millipore, Prague, Czech Republic, conductivity < 0.05 μS cm^−1^).

### 2.3. Apparatus

The voltammetric signals were recorded by PC ETP (Polaro-Sensors, Prague, Czech Republic) controlled by MultiElChem 3.3 software (J. Heyrovský Institute of Physical Chemistry, Prague, Czech Republic). A three-electrode system was used in all cases with Ag|AgCl|KCl(sat.) as the reference electrode (Monokrystaly, Turnov, Czech Republic) and platinum plate (1 cm^2^) auxiliary electrode. As the working electrode, three types of electrodes were used: (a) the hanging mercury drop electrode (HMDE, Polaro Sensors, Prague, Czech Republic), drop surface area 1.365 mm^2^; (b) the glassy carbon electrode (GCE, laboratory-made), disc diameter 3.0 mm, surface area 7.1 mm^2^, polished on alumina suspension between measurements (particle size 1.1 µm, Elektrochemické detektory, Turnov, Czech Republic); and (c) the laboratory-made electrode from Carbon Fiber PLA (CF-PLA, ProtoPlant, WA, USA), diameter 1.75 mm, surface area 2.4 mm^2^, with sides insulated by shrinking rubber tube, polished on the sanding paper K/400 (Ciret, Havant, United Kingdom) [4].

Electrochemical pretreatment of GCE and CF-PLA [8,25,26,27,28] was carried out in 3 mol L^−1^ KCl before each voltammetric experiment, and parameters were set individually for each used electrode type. At GCE, 150 cycles were applied, switching every 0.1 s between potentials 100 mV more negative than the onset of hydrogen evolution reaction (*E*_Clean_,_neg_) and 100 mV more positive than the potential of oxygen evolution or material dissolution (*E*_Clean_,_pos_), always ending at *E*_Clean_,_neg_. At CF-PLA, 10 cycles of CV between *E*_Clean_,_neg_, and *E*_Clean_,_pos_ were applied with a scan rate of 50 mV s^−1^ [30,32,33]. No pretreatment was applied at the HMDE.

Statistical parameters were calculated using QC Expert software 3.1 (Trilobyte, Pardubice, Czech Republic). Critical level (C*_c_*) was calculated according to IUPAC recommendations as C*_c_* = 1.645 σ_B_, where σ_B_ is the standard deviation of the blank noise (*n* = 5) [23].

### 2.4. Procedures

The 3D printed models were immersed in 20 mL of the particular tested solvent for 24 h under the ambient temperature. After that time, the solvents were used for voltammetric experiments; measurements were performed in the supporting electrolytes without and with the addition of Cd^2+^ (1 × 10^–3^ mol dm^−3^), [Ru(NH_3_)_6_]^3+^ (1 × 10^–3^ mol dm^−3^) or [Fe(CN)_6_]^3^^–^ (1 × 10^–4^ mol dm^−3^). The solutions were bubbled for 10 min with nitrogen before an experiment with a nitrogen atmosphere over the solution during an experiment. Cyclic voltammetry (CV) was performed with a scan rate of 50 mV s^−1^. Differential pulse voltammetry (DPV) used a scan rate of 20 mV s^−1^, a pulse width of 100 ms, and a pulse height of 50 mV. Anodic stripping differential pulse voltammetry (DPASV) was used only for Cd^2+^ determination with a scan rate of 20 mV s^−1^, pulse width 100 ms, pulse height 50 mV, and accumulation time 15 s; the accumulation potential varied according to the used solvent and electrode, as summarized in Table 1. All current values were recalculated to current densities (*J*) or peak current densities (*J*_p_) to compare the registered signals.

### 2.5. Surface Imaging

The tested 3DPMs objects were inspected by optical microscopy before and after the immersion to selected solvents. The square plates were investigated by the optical microscope (DigiMicroLab 5.0, DNT, Leer, Germany) and NMM800TR Transmitting & Reflecting Metallurgical Microscope (Microteb, Teheran, Iran). Images were taken by Dino-Eye AM4023CT USB C-Mount Microscope Camera (Dino-Lite Digital Microscopes, AnMo Electronics Corporation, New Taipei City, Taiwan) with a magnification of 50×.

## 3. Results and Discussion

### 3.1. Effect of Organic Solvents and Supporting Electrolytes on Mechanical and Physical Properties

Initially, the experiments were aimed at the effects of various liquids (organic solvents and inorganic or mixed aqueous–organic electrolytes) on the mechanical and physical stability of 3DPMs objects, including their swelling, which can cause serious distortion of printed objects. Some of the chemical incompatibilities were already studied elsewhere, notably PET in acetone and ethanol; and PLA in various solutions [21,31] under applied mechanical pressure. Our results were fully in agreement, even without the application of the external force. Notably, PLA was delaminated in acetone, and PET was swelled and softened. We have included these experiments anyway for clarity and direct comparison with the other results using additional 18 types of solvents and solutions.

Table 2 summarizes the observed changes of the 3DPMs after 24-h exposure to the selected solvents, including swelling-induced expansion, dissolution, or delamination.

We can see that objects printed from Elastic resin show high stability towards the organic solvents. On the other hand, it suffers from excessive swelling; in some cases, it increases its volume by 50%.

Clear resin and PET showed similar stability. However, they are, to a different extent, vulnerable to chloroform, dichlormethane, tetrahydrofuran, N,N-dimethylformamide, and acetone; the observed swelling is generally accompanied by some other damage. In the case of Clear resin, the breaking seems to be caused by the tension of the irregular swelling; PET is more susceptible to dissolution.

The most significant effect was observed in the case of PLA—half of the solvents caused the dissolution or deformation caused by the delamination of the layers. Although the PLA is a widely available and relatively cheap material, its incompatibility with many commonly used solvents is a significant drawback for some applications.

Equally important, chlorinated solvents are most likely to cause damage to the printing materials, followed by other polar aprotic solvents, e.g., tetrahydrofuran, acetone, and acetonitrile.

Optical microscopy was used to observe the 3DPMs after the immersion in the electrolytes. Some level of disruption was found on all materials after exposition to NaOH solution; in the case of PLA, the damage was macroscopically visible. Clear PET and PLA were also damaged by the solution containing methanol, and PLA by the H_2_SO_4_ solution. The complete optical microscopy images are available in the Supplementary Materials as Figures S1–S4.

### 3.2. Effect of Materials Exposure on the Electrochemical Properties of Electrolytes

The effects of 24-h exposure of the tested 3DPMs on the electrochemical properties of selected electrolytes were investigated by CV. We monitored the presence of electrochemically active interferences relased from the printed objects, and the influence of other species on the potential window, e.g., via hydrogen evolution reaction, oxygen evolution reaction, or by dissolving the 3D printed electrode material.

**Figure 2:**
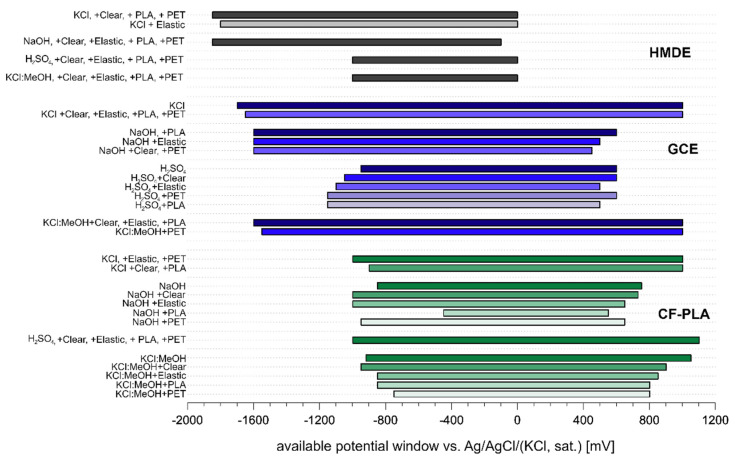
Available potential windows in various electrolytes before and after their 24-h exposition to 3D-printed objects from Elastic, Clear, PET, and PLA at HMDE, GCE, and CF-PLA.

The anodic or cathodic dissolution/solvent decomposition onset potentials at the end of a potential window (*E*_End_) were set, when the registered current reached a chosen level (*I*_End_), set according to the electrode area and background current, with a primary objective to compare potential window changes at a particular electrode type. For GCE, the cathodic *I*_End_ was −50 µA and the anodic *I*_End_ was 40 µA; for HMDE, the cathodic *I*_End_ was −2.0 µA and −10 µA and the anodic *I*_End_ was 0.5 and 2.0 µA in aqueous solutions and mixed organic–aqueous solution, respectively; for CF-PLA, the cathodic *I*_End_ was −2.0 and −20 µA and anodic *I*_End_ was 0.5 and 20 µA in aqueous solutions and mixed organic aqueous solution, respectively. Found potential window widths are shown in Figure 2.

Generally, it is possible to conclude that potential windows before and after exposure to 3DPMs were similar. In the case of HMDE, no significant difference was observed. The changes in the available GCE potential window are mostly negligible except for H_2_SO_4,_ where depolymerization after H^+^ attack of PLA [34] and PET led to a rise in pH and the consequent shift of the potential window. A slightly narrower potential window is also observed in NaOH solution after exposure to PET and Clear resin.

CF-PLA exhibits the most distinct difference in the potential window width. Furthermore, narrower potential windows and higher charging currents on CF-PLA were observed in mixed organic–aqueous media. This behavior suggests that the CF-PLA electrode surface was affected by the solvents, dissolving the PLA and thus exposing more electrically conductive carbon fiber, leading to the increase of registered currents. A similar effect was described and employed for a 3D printed electrode activation by Browne et al. [28]. Besides that, a significant change in the potential window width was registered in NaOH solution after the exposure to PLA.

### 3.3. Effect of Exposure of Supporting Electrolytes to 3DPMs on Voltammetric Behavior of Selected Redox Systems

DPV of the selected simple and well-known redoxactive probes was performed using all three tested electrodes to monitor changes in the voltammetric response after exposure of the selected solvents to 3DPMs. As the selected analytes, [Ru(NH_3_)_6_]^3+^ was employed for all the used electrodes; [Fe(CN)_6_]^3^^–^ was used for the electrodes with wide anodic potential range (i.e., GCE and CF-PLA); whilst at HMDE, DPV, and DPASV analyses of Cd^2+^ ions were performed instead. The 0.1 mol dm^−3^ NaOH solution was omitted due to the low solubility of Cd^2+^ ions. The results are summarized in Table 3 (HMDE), Table 4 (GCE), and Table 5 (CF-PLA).

Despite the low level of macroscopically visible interaction of the solvents with the 3DPMs, the electrochemical interference was quite serious. The observed interferences occur primarily in the solvents, which caused mechanical damage as well, namely methanol-containing solution and NaOH solution, partly H_2_SO_4_ solution. The signals observed in the KCl solution were not influenced by the presence of any 3DPMs.

Contrary to the stability of 3DPMs, the main source of the interferences is Elastic resin; distinct peak distortion or even its disappearance was observable in most of the solvents. Moreover, this material releases electrochemically active compounds. In Clear resin, the presence of undesired compounds was also observed in methanol-containing solution; otherwise, this material interfered with the analyte signals only rarely.

The suppression of the analyte signals is also electrode dependent—it is most pronounced at HMDE, probably because of the specific sensitivity of the metallic surface. Results obtained using the CF-PLA electrode were somewhat scattered considering the peak height and potential, probably due to the interference of the electrolyte with the electrode material.

## 4. Conclusions

The research focused on the stability of four different 3DPMs (Clear and Elastic resins for stereolitography, and FMD filaments PLA and PET) in various organic solvents and how they affect the voltammetric behavior of selected redox probes at various electrodes if the material is in contact with the electrolyte solution.

Elastic resin did not suffer mechanical damage in contact with any of the tested solvents, although it underwent severe swelling. The reason might also lay in its flexibility, which protected it during the swelling—the mechanical damage caused to Clear resin seemed to be caused by the irregular swelling in some of the solvents. PET and PLA suffered more from delamination and dissolution; PLA to a greater extent than PET.

Potential window width was only slightly influenced by the contact with the 3DPMs. The only exception was the CF-PLA electrode which might be caused by the interaction of the electrolyte with the electrode material. On the other hand, the exposure of the 3DPMs to selected supporting electrolytes can influence the voltammetric behavior of analytes and suppress their faradaic response; this suppression is electrode-dependent. Elastic resin releases the most active interferences, although its outer appearance is not changed. The main finding achieved in this work is that even seemingly stable materials might not be suitable for the electrochemical application. The objects printed from Clear resin were mostly stable with the least amount of electrochemically interfering properties, and their suitability for the fabrication of 3D-printed electrochemical cells is highest, but their stability in strongly basic or mixed aqueous–organic solvents is also limited. The obtained results underline the necessity of a careful approach to the application of these materials in demanding conditions.

## Figures and Tables

**Table 1:** Accumulation potentials in various solvents used during DPASV measurement.

	Electrode:	HMDE	GCE	CF-PLA
Solvent		Accumulation potential (*E*_acc_) [mV]		
1 mol dm^−3^ KCl, 1 mol dm^−3^ NaOH		−1800	−1600	−1000
1 mol dm^−3^ KCl: MeOH (9:1, *v/v*)		−1000	−1600	−1000
1 mol dm^−3^ H_2_SO_4_		−1000	−1000	−1000

**Table 2:** Changes of 3D printed square plate objects (10 × 10 × 1 mm) after 24-h immersion to various solvents and solutions. Numbers denote relative elongation (%) of the objects after the immersion.

Solvent	Material			
	Elastic	Clear	PET	PLA
Acetone	25	7 cracked	× soft	13 delamination
Acetonitrile	14	2	×	17 delamination
Chloroform	53	--- broken	--- dissolved	--- dissolved
Dichloromethane	45	16 broken	--- dissolved	--- dissolved
Diethyl ether	21	×	×	×
Dimethyl sulfoxide	24	×	× soft	5 soft
Ethanol	33	2	×	×
Ethyl acetate	29	×	× soft	12 cracked, soft
Formaldehyde 38%	7	×	×	×
Hexane	5	×	×	×
Isopropanol	29	×	×	×
Methanol	24	8 cracked	×	×
N,N-dimethylformamide	38	3 deformation	3 soft, deformation	23 delamination
n-penthanol	32	×	×	×
Tetrahydrofuran	46	4 deformation	--- partly dissolved	--- dissolved
Toluene	33	×	×	8 cracked, soft,
Aqueous 1M KCl	×	×	×	×
Aqueous 1M NaOH	×	×	×	etched
Aqueous 1M H_2_SO_4_	×	×	×	×
1M KCl: MeOH (1:9, *v*/*v*)	19	6	×	×

× no observed change; --- impossible to obtain the value.

**Table 3:** DPV and DPASV signals of Cd^2+^ and [Ru(NH_3_)_6_]^3+^ recorded using HMDE before and after 24-h electrolyte exposures to 3DPMs.

Printing Material		Before	Elastic	Clear	PET	PLA
Supporting electrolyte		1 mol dm^−3^ KCl				
Cd^2+^; DPV	*E*_p_ [mV]	−589	−584	−589	−586	−589
	*J*_p_ [µA mm^−2^]	1.16	1.09	1.32	1.21	1.16
Cd^2+^; DPASV	*E*_p_ [mV]	−587	−580	−584	−582	−592
	*J*_p_ [µA mm^−2^]	2.78	1.90	2.12	1.99	2.21
[Ru(NH_3_)_6_]^3+^; DPV	*E*_p_ [mV]	−154	−145	−151	−148	−149
	*J*_p_ [µA mm^−2^]	0.18	0.19	0.17	0.18	0.22
Supporting electrolyte		1 mol dm^−3^ NaOH				
[Ru(NH_3_)_6_]^3+^; DPV	*E*_p_ [mV]	−238	-	−229	-	-
	*J*_p_ [µA mm^−2^]	0.18	<C*_c_*	0.04	<C*_c_*	<C*_c_*
Supporting electrolyte		1 mol dm^−3^ H_2_SO_4_				
Cd^2+^; DPV	*E*_p_ [mV]	−541	-	−541	−546	−540
	*J*_p_ [µA mm^−2^]	0.94	<C*_c_*	0.43	0.43	0.46
Cd^2+^; DPASV	*E*_p_ [mV]	−587	−493	−589	−589	−588
	*J*_p_ [µA mm^−2^]	2.15	0.28	2.10	1.79	2.85
[Ru(NH_3_)_6_]^3+^; DPV	*E*_p_ [mV]	−180	−173	−177	−176	−176
	*J*_p_ [µA mm^−2^]	0.22	0.22	0.21	0.21	0.23
Supporting electrolyte		1 mol dm^−3^ KCl: MeOH (1:9, *v/v*)				
Cd^2+^; DPV	*E*_p_ [mV]	−714	-	−704	−726	−722
	*J*_p_ [µA mm^−2^]	0.59	<C*_c_*	0.68	2.98	2.59
Cd^2+^; DPASV	*E*_p_ [mV]	−647	−639	−636	−636	−659
	*J*_p_ [µA mm^−2^]	1.57	0.32	0.10	0.14	1.11
[Ru(NH_3_)_6_]^3+^; DPV	*E*_p_ [mV]	−165	−171	−153	−159	−159
	*J*_p_ [µA mm^−2^]	0.20	0.06	0.17	0.23	0.24

C*_c_*—Critical level.

**Table 4:** Evaluation of DPV and DPASV signals of Cd^2+^, [Ru(NH_3_)_6_]^3+^, and [Fe(CN)_6_]^3−^ recorded using GCE before and after 24-h electrolyte exposures to 3DPMs.

Printing Material		Before	Elastic	Clear	PET	PLA
Supporting electrolyte		1 mol dm^−3^ KCl				
Cd^2+^; DPV	*E*_p_ [mV]	–589	–584	–589	–586	–589
	*J*_p_ [µA mm^−2^]	1.16	1.09	1.32	1.21	1.16
Cd^2+^; DPASV	*E*_p_ [mV]	–587	–580	–584	–582	–592
	*J*_p_ [µA mm^−2^]	2.78	1.90	2.12	1.99	2.21
[Ru(NH_3_)_6_]^3+^; DPV	*E*_p_ [mV]	–154	–145	–151	–148	–149
	*J*_p_ [µA mm^−2^]	0.18	0.19	0.17	0.18	0.22
[Fe(CN)_6_]^3−^	*E*_p_ [mV]	233	233	232	230	229
	*J*_p_ [µA mm^−2^]	2.04	2.10	2.24	2.20	2.26
Supporting electrolyte		1 mol dm^−3^ NaOH				
[Ru(NH_3_)_6_]^3+^; DPV	*E*_p_ [mV]	–238	-	–229	-	-
	*J*_p_ [µA mm^−2^]	0.18	<C*_c_*	0.04	<C*_c_*	<C*_c_*
Supporting electrolyte		1 mol dm^−3^ H_2_SO_4_				
Cd^2+^; DPV	*E*_p_ [mV]	–541	-	–541	–546	–540
	*J*_p_ [µA mm^−2^]	0.94	<C*_c_*	0.43	0.43	0.46
Cd^2+^; DPASV	*E*_p_ [mV]	–587	–493	–589	–589	–588
	*J*_p_ [µA mm^−2^]	2.15	0.28	2.10	1.79	2.85
[Ru(NH_3_)_6_]^3+^; DPV	*E*_p_ [mV]	–180	–173	–177	–176	–176
	*J*_p_ [µA mm^−2^]	0.22	0.22	0.21	0.21	0.23
[Fe(CN)_6_]^3−^	*E*_p_ [mV]	271	263	264	268	268
	*J*_p_ [µA mm^−2^]	2.00	1.94	2.04	1.84	1.92
Supporting electrolyte		1 mol dm^−3^ KCl: MeOH (1:9, *v/v*)				
Cd^2+^; DPV	*E*_p_ [mV]	–714	-	–704	–726	–722
	*J*_p_ [µA mm^−2^]	0.59	<C*_c_*	0.68	2.98	2.59
Cd^2+^; DPASV	*E*_p_ [mV]	–647	–639	–636	–636	–659
	*J*_p_ [µA mm^−2^]	1.57	0.32	0.10	0.14	1.11
[Ru(NH_3_)_6_]^3+^; DPV	*E*_p_ [mV]	–165	–171	–153	–159	–159
	*J*_p_ [µA mm^−2^]	0.20	0.06	0.17	0.23	0.24
[Fe(CN)_6_]^3−^	*E*_p_ [mV]	154	129	179	116	163
	*J*_p_ [µA mm^−2^]	0.17	0.04	0.22	0.14	0.12

C*_c_*—Critical level.

**Table 5:** Evaluation of DPV of [Fe(CN)_6_]^3^^–^ and [Ru(NH_3_)_6_]^3+^ recorded using CF-PLA before and after 24-h electrolyte exposures to 3DPMs.

Printing Material		Before	Elastic	Clear	PET	PLA
Supporting electrolyte		1 mol dm^−3^ KCl				
[Ru(NH_3_)_6_]^3+^; DPV	*E*_p_ [mV]	–158	–163	–158	–158	–158
	*J*_p_ [µA mm^−2^]	0.138	0.093	0.097	0.129	0.130
[Fe(CN)_6_]^3^^–^; DPV	*E*_p_ [mV]	519	439	439	413	435
	*J*_p_ [µA mm^−2^]	0.002	0.004	0.002	0.002	0.002
Supporting electrolyte		1 mol dm^−3^ NaOH				
[Ru(NH_3_)_6_]^3+^; DPV	*E*_p_ [mV]	–189	–192	–197	–203	–180
	*J*_p_ [µA mm^−2^]	0.059	0.030	0.052	0.066	0.051
[Fe(CN)_6_]^3^^–^; DPV	*E*_p_ [mV]	339	240	234	242	229
	*J*_p_ [µA mm^−2^]	0.007	0.131	0.133	0.069	0.111
Supporting electrolyte		1 mol dm^−3^ H_2_SO_4_				
[Ru(NH_3_)_6_]^3+^; DPV	*E*_p_ [mV]	–198	–204	–201	–201	–223
	*J*_p_ [µA mm^−2^]	0.037	0.023	0.022	0.023	0.022
[Fe(CN)_6_]^3^^–^; DPV	*E*_p_ [mV]	237	304	303	256	296
	*J*_p_ [µA mm^−2^]	0.014	0.011	0.009	0.011	0.010
Supporting electrolyte		1 mol dm^−3^ KCl: MeOH (1:9, *v/v*)				
[Ru(NH_3_)_6_]^3+^; DPV	*E*_p_ [mV]	–141	–250	–149	–170	–170
	*J*_p_ [µA mm^−2^]	0.517	0.631	0.502	0.577	0.415
[Fe(CN)_6_]^3^^–^; DPV	*E*_p_ [mV]	299	53	107	-	-
	*J*_p_ [µA mm^−2^]	0.005	0.183	0.048	<C*_c_*	<C*_c_*

C*_c_*—Critical level.

## Data Availability

Not applicable.

## Supplementary Materials

The following are available online at https://www.mdpi.com/article/10.3390/bios12050308/s1, Figure S1: Optical microscopic and transmitting & reflecting microscopic images of 3D printing material PET before and after 24-h exposure to electrolytes), Figure S2: Optical microscopic and transmitting & reflecting microscopic images of 3D printing material PLA before and after 24-h exposure to electrolytes, Figure S3: Optical microscopic and transmitting & reflecting microscopic images of 3D printing material Elastic before and after 24-h exposure to electrolytes, Figure S4: Optical microscopic and transmitting & reflecting microscopic images of 3D printing material Clear before and after 24-h exposure to electrolytes.
