# Supplementary material for: Applicability of Selected 3D Printing Materials in Electrochemistry
Source: Biosensors (Basel). 2022 May 7;12(5):308. doi: 10.3390/bios12050308 (PMC9138249; doi:10.3390/bios12050308)
Supplement: Supplementary file 1 [file biosensors-12-00308-s001.zip › biosensors-1700537-supplementary.pdf]

biosensors

MDPI

Article

Applicability of Selected 3D Printing Materials in Electrochemistry

Marta Choińska [1,2], Vojtěch Hrdlička [1], Hana Dejmková [2], Jan Fischer [2], Luděk Míka [3], Eva Vaněčková [1], Viliam Kolivoška [1] and Tomáš Navrátil [1,*]

[1] J. Heyrovský Institute of Physical Chemistry of the Czech Academy of Sciences, Dolejškova 3, 182 23 Prague, Czech Republic; marta.choinska@jh-inst.cas.cz (M.C.); vojtech.hrdlicka@jh-inst.cas.cz (V.H.); eva.vaneckova@jh-inst.cas.cz (E.V.); viliam.kolivska@jh-inst.cas.cz (V.K.)

[2] Department of Analytical Chemistry, Faculty of Science, Charles University,Albertov 6, 128 00 Prague, Czech Republic; hana.dejmkova@natur.cuni.cz (H.D.); jan.fischer@natur.cuni.cz (J.F.)

[3] Department of Chemistry Education, Faculty of Science, Charles University, Albertov 6, 128 00 Prague, Czech Republic; ludek.mika@natur.cuni.cz

* Correspondence: tomas.navratil@jh-inst.cas.cz; Tel.: +420-266-051-111

Citation: Choińska, M.; Hrdlička, V.; Dejmková, H.; Fischer, J.; Míka, L.; Vaněčková, E.; Kolivoška, V.; Navrátil, T. Applicability of Selected 3D Printing Materials in Electrochemistry. *Biosensors* **2022**, *12*, 308. https://doi.org/10.3390/bios12050308

Received: 11 April 2022
Accepted: 30 April 2022
Published: 7 May 2022

Publisher's Note: MDPI stays neutral with regard to jurisdictional claims in published maps and institutional affiliations.

Copyright: © 2022 by the authors. Licensee MDPI, Basel, Switzerland. This article is an open access article distributed under the terms and conditions of the Creative Commons Attribution (CC BY) license (https://creativecommons.org/licenses/by/4.0/).

Figures S1–S4 were obtained by optical microscopy before and after the immersion of 3DPMs to selected solvents. The square plates were investigated by the optical microscope (DigiMicroLab 5.0, DNT, Germany) and NMM800TR Transmitting & Reflecting Metallurgical Microscope (Microteb, Iran). Images were taken by Dino-Eye AM4023CT USB C-Mount Microscope Camera (Dino-Lite Digital Microscopes, AnMo Electronics Corporation, Taiwan) with 50x magnification.

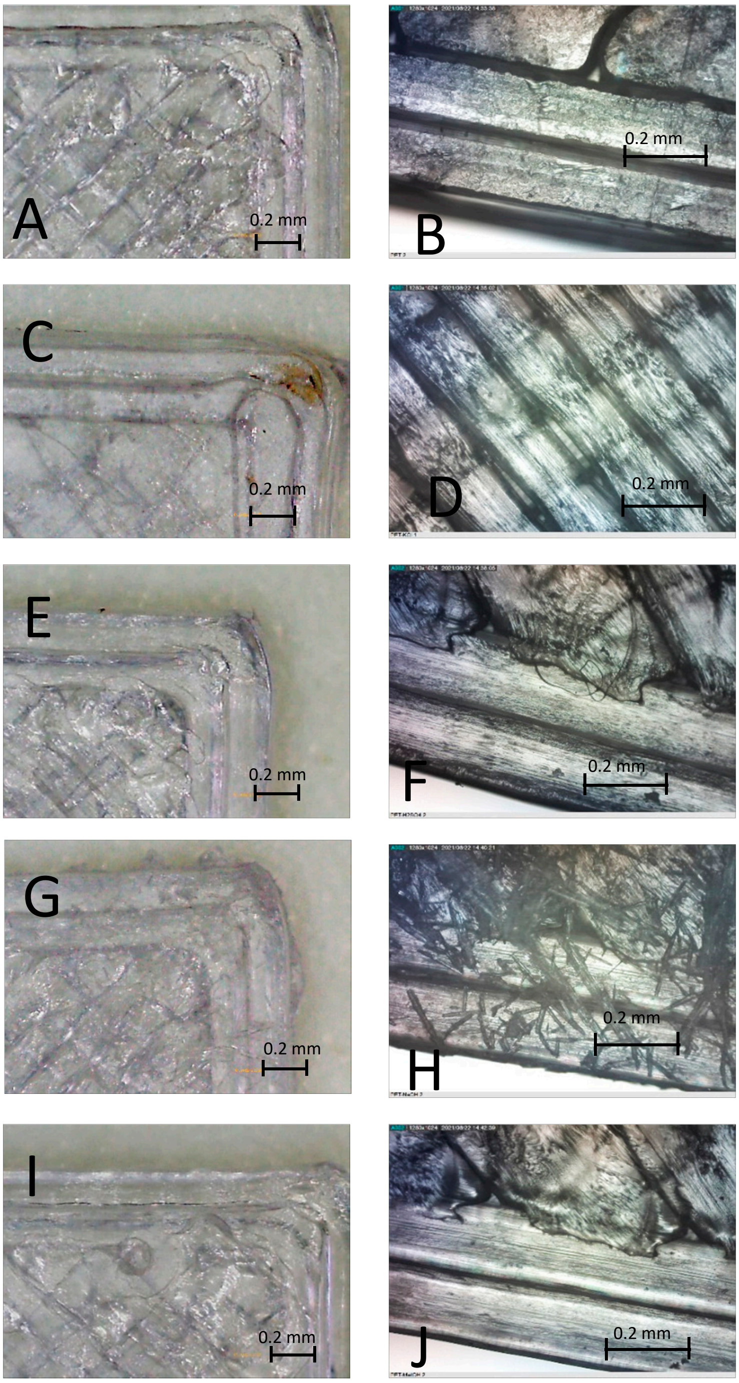

Figure S1. Optical microscopic (**A, C, E, G, I**) and transmitting & reflecting microscopic (**B, D, F, H, J**) images of 3D printing material **PET** before (**A, B**) and after 24-hours exposure to electrolytes: (**C, D)**: 1 mol dm^{-3} KCl; (**E, F**): 1 mol dm^{-3} H_2SO_4; (**G, H)** – 1 mol dm^{-3} NaOH; (**I, J)** - methanol mixed with 1 mol dm^{-3} KCl (9:1).

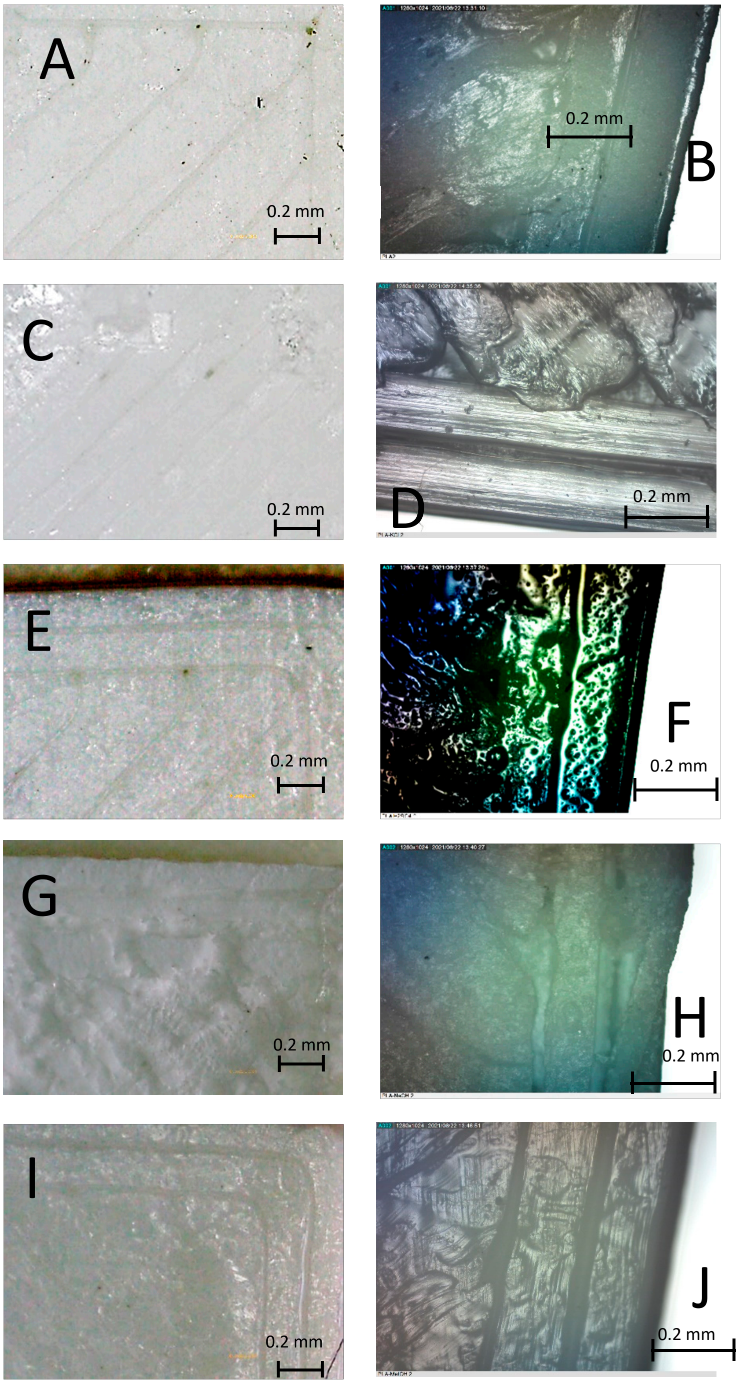

Figure S2. Optical microscopic (**A, C, E, G, I**) and transmitting & reflecting microscopic (**B, D, F, H, J**) images of 3D printing material **PLA** before (**A, B**) and after 24-hours exposure to electrolytes: (**C, D**): 1 mol dm^{-3} KCl; (**E, F**): 1 mol dm^{-3} H_2SO_4; (**G, H)** – 1 mol dm^{-3} NaOH; (**I, J)** - methanol mixed with 1 mol dm^{-3} KCl (9:1).

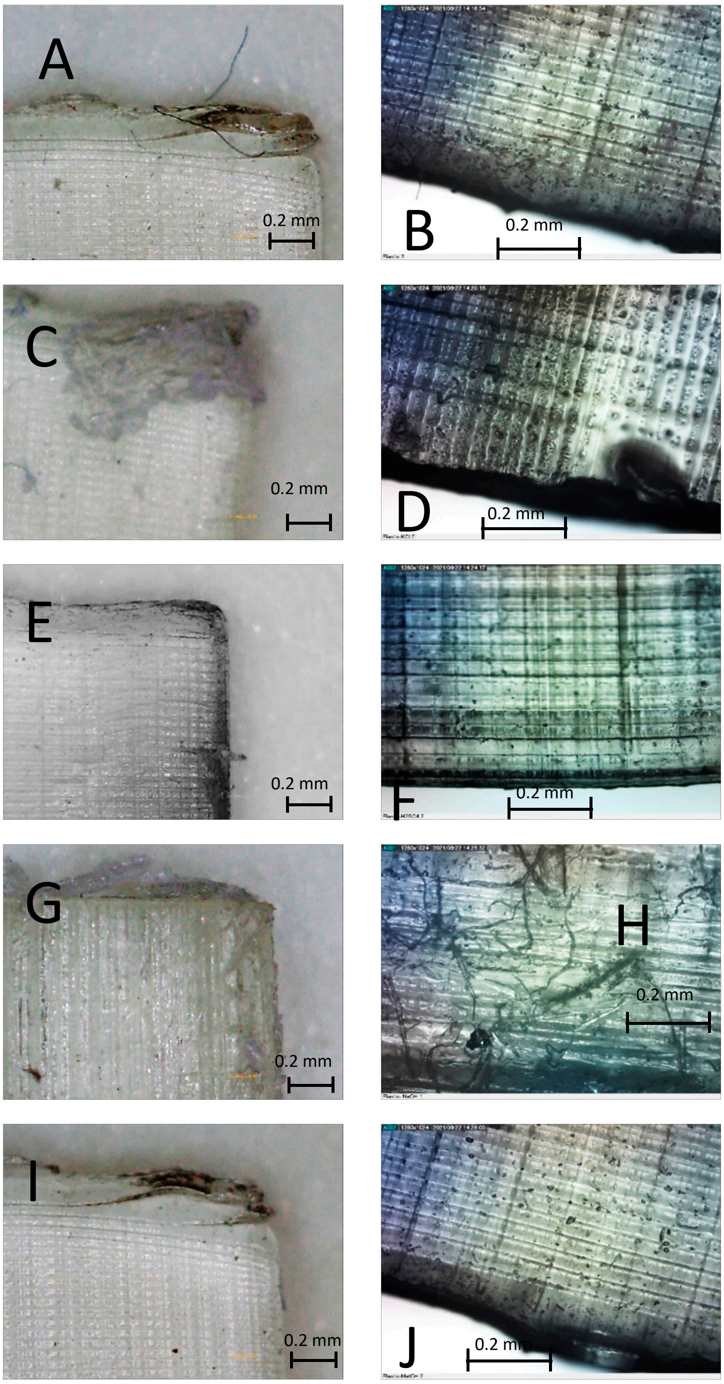

Figure S3. Optical microscopic (**A, C, E, G, I**) and transmitting & reflecting microscopic (**B, D, F, H, J**) images of 3D printing material **Elastic** before (**A, B**) and after 24-hours exposure to electrolytes: (**C, D**): 1 mol dm^{-3} KCl; (**E, F**): 1 mol dm^{-3} H_2SO_4; (**G, H**) – 1 mol dm^{-3} NaOH; (**I, J**) - methanol mixed with 1 mol dm^{-3} KCl (9:1).

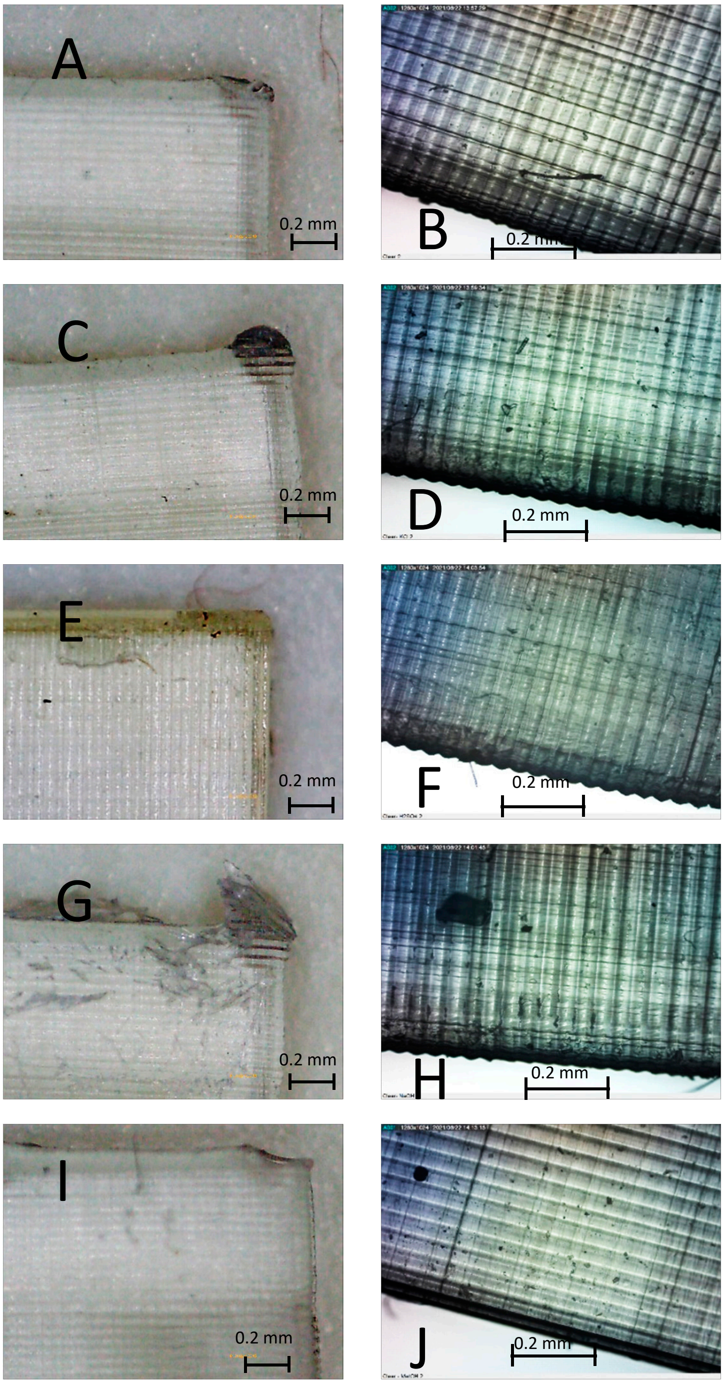

Figure S4 Optical microscopic (**A, C, E, G, I**) and transmitting & reflecting microscopic (**B, D, F, H, J**) images of 3D printing material **Clear** before (**A, B**) and after 24-hours exposure to electrolytes: (**C, D**): 1 mol dm^{-3} KCl; (**E, F**): 1 mol dm^{-3} H_2SO_4; (**G, H**) – 1 mol dm^{-3} NaOH; (**I, J**) - methanol mixed with 1 mol dm^{-3} KCl (9:1).
